# COVID-19 and Traumatic Brain Injury (TBI); What We Can Learn From the Viral Pandemic to Better Understand the Biology of TBI, Improve Diagnostics and Develop Evidence-Based Treatments

**DOI:** 10.3389/fneur.2021.752937

**Published:** 2021-12-20

**Authors:** Denes V. Agoston

**Affiliations:** Department of Anatomy, Physiology and Genetics, Uniformed Services University, Bethesda, MD, United States

**Keywords:** COVID-19, TBI, diseases, definitions, lessons, applications, pandemics epidemics

Traumatic brain injury (TBI) has been referred to as the “silent epidemic” but given its substantial and recurring impact on global health, it should be renamed to the “silent pandemic”. The COVID-19 pandemic has majorly impacted biomedical research of TBI; experimental and clinical studies have either slowed or halted and resources have been reallocated, thus resulting in a “lost year” for the TBI field. However, the pandemic can serve as an inflection point. The conceptual and technical lessons that can be learned from COVID-19 and could lead to clearer classification, improved diagnosis, and potentially improved treatment efficacy of TBI are presented in this paper.

## Introduction

These are very challenging times for all mankind as severe acute respiratory syndrome coronavirus 2 (SARS-CoV-2) has been infecting the entire global population. There are already lessons learned, and even more to come after the pandemic ends, with profound effects on virtually every aspect of our lives, the economy, work, social life, and health care. Major crises, wars, and pandemics have historically forced humanity to learn, adapt, and come up with solutions by developing new technologies, business models, and even entire industries. They have also spurred major advances in biology through better understanding of diseases, identification of pathogens and their pathomechanisms, and development of novel treatments. The same is happening now during the COVID-19 pandemic, as the scientific progress is astonishing.

Within less than a year, the pathogen SARS-CoV-2 has been isolated, its genome sequenced, and the pathomechanism of coronavirus disease 2019 (COVID-19) has been identified (1, 2). Less than a year after China shared the genetic sequence of the virus (January 12, 2020) (2), an astonishing number of vaccine candidates have been in various phases of development (https://covid-19tracker.milkeninstitute.org; https://www.who.int/publications/m/item/draft-landscape-of-covid-19-candidate-vaccines). Merely 11 months later, on December 11, 2020, the FDA authorized the first vaccine developed by BioNTech (in partnership with Pfizer) that showed ~95% efficacy. Within a year of the outbreak, millions have already been vaccinated with the BioNTech/Pfizer and Moderna vaccines. This astonishingly rapid progress is due to many factors, “lessons learned” from previous epidemics, better understanding of human health and disease processes, clear understanding the pathobiology of COVID-19, identifying targets for vaccines and for pharmacological and biological treatments and critically by using a revolutionary, new mRNA-based technology.

Furthermore, there are hundreds of treatments for COVID-19 patients in various phases of clinical trials, many of which are repurposed drugs (https://covid-19tracker.milkeninstitute.org/#treatment). Among these drugs are antivirals (e.g., Remdesivir), monoclonal antibody cocktails (e.g., REGN-COV2), and anti-inflammatory drugs that are either in clinical trial or have received emergency use authorization (https://clinicaltrials.gov/ct2/show/NCT04350593 and https://www.pnas.org/content/117/44/27141 and https://science.sciencemag.org/content/369/6508/1261 and https://www.eurekalert.org/pub_releases/2020-11/rci-dnp110420.php).

While the world understandably focuses on the COVID-19 pandemic, other diseases have not disappeared. TBI affects ~3 million people, kills ~50,000, and permanently disables ~80,000 people in the US alone EVERY *year*. (https://www.cdc.gov/traumaticbraininjury/get_the_facts.html' August 31, 2020). Worldwide numbers are roughly an order of magnitude higher and are rapidly rising especially in developing countries (3, 4). Based on these numbers alone, TBI should be called “silent pandemic” that occurs year after year!

This Opinion article list some of the lessons that can be learned from COVID-19 and can be apply some of them in TBI research. Some of the potential lessons are conceptual, some are technical.

## Conceptual

### Definition

Defining COVID-19 only as an “infection” or even a “viral infection” would be an oversimplification. Like SARS-CoV-2, the infectious agent, TBI is not the disease itself. It is only a causative event that can be either prevented or its consequences mitigated. TBI causes structural perturbance to the brain and/or damage at the molecular, cellular, and regional level of varying severity followed by pathobiological responses that determine the disease phenotype. It may seem like a matter of semantics but using the term TBI to describe the disease can lead to an overly simple thought process. Since there is no drug for treating an “infection” *per se*, there is and will be no drug for “treating TBI”.

### Spectrum of TBI

SARS-CoV-2 infection causes a spectrum of disease severities from mild, flu-like symptomatology to severe viral pneumonia (5). Despite its scariness and harrowing toll, even severe COVID-19 is a relatively simple disease phenotype compared to the disease phenotypes caused by TBI. Different types and intensities of physical forces cause different types and severities of brain injury that precipitate in varying degrees of neuropsychological/functional impairments, traditionally classified as mild, moderate, and severe. The combination of the initial physical damage and the ensuing pathobiological responses result in different disease phenotypes that are characterized by distinct dominant pathologies (6). These make TBI several orders of magnitude more complex than COVID-19. So how do we deal with such complexity? As in the case of infectious diseases where the pathogens as well as their pathomechanisms are identified, in addition to the type of primary injury, we must be able to identify the pathobiological process triggered by the initial injury.

### Diagnosis

Images of people with thermal readers, the “gun-looking” devices, have become familiar scenes during COVID-19. Elevated body temperature can indicate that an individual has COVID-19 but they could also be suffering from dozens of other infectious diseases. With respect to TBI, we are currently only “checking body temperatures.” Injury markers such as ubiquitin carboxy-terminal hydroxylase 1 (UCH-L1), glial fibrillary acidic protein (GFAP), and neurofilament light chain (NF-L) are excellent and sensitive indicators of neuron and glia injury (7) but single post-injury time point data greatly limit their full diagnostic potential (8). They however do not provide information about the underlying pathobiological changes and cannot be used to identify disease phenotype(s). Like COVID-19 diagnostic testing, “mechanistic” biochemical markers (e.g., inflammatory, metabolic) need to be added to the current panel of damage markers so we can identify the pathobiological responses to TBI. These biomarkers will be critical for identifying disease (endo)phenotype(s) (9) and guiding specific, targeted treatments (10). However, lessons learned from COVID-19 should change how we use and interpret existing biomarker data (11). COVID-19 has majorly altered the clinical trial landscape in TBI as in many other disorders and not only from the logistical and organizational perspective. The patient population in the post-COVID-19 era will be different in many ways and will present new challenges for diagnosis and prognosis in TBI.

### Temporal Aspect

Clinical studies have shown that treatment with antivirals (e.g., Remdesivir) is only effective during the early phase (7-10 days) of infection (12, 13) while anti-inflammatory treatments (e.g., dexamethasone or anti-IL-6 monoclonals) do more harm than good during this phase (14, 15). Conversely, anti-inflammatory treatment can significantly reduce unfavorable outcome and death at the later phase of the disease. Inflammation is a key adaptive response to any kind of noxious stimuli in all multicellular organisms (16–18). Neuroinflammation has been extensively studied since the 1990s but all experimentally successful anti-inflammatory treatments have failed in clinical TBI trials (19). This can be plausibly explained by the lack of key information regarding the temporal pattern of the inflammatory process, and its specific components that cannot be determined by using markers of damage. We know that there is a neuroinflammatory response to TBI (20) but the temporal pattern of its different phases, reparative vs. injurious, and their cellular and humoral components are currently not well known. It is likely that the many experimentally successful pharmacological treatments, including anti-inflammatories, missed their therapeutic windows in clinical trials, an issue compounded by inter-species differences and flaws in experimental design. We know that time is a critical dimension of most disease processes, including TBI, so we must identify not only the pathobiological responses after TBI but also their temporal patterns; both are essential for developing specific treatments and applying them within their therapeutic window to successfully mitigate the consequences of TBI.

### Systemic Nature of TBI

COVID-19 mainly involves the respiratory system but like most diseases, it is multi-systemic, it can affect all systems from gastrointestinal to the CNS (5, 21). Clinical and especially experimental TBI studies have almost exclusively focused on the brain, largely ignoring the rest of the body. This is based on the traditional view of the blood brain barrier (BBB) as a “brick wall” with binary function: closed or open. There is already ample evidence about the complexities of BBB function, from regulating transendothelial transport to real “opening” (22). Endothelial stress and microvascular injury are emerging as important, maybe even dominant, (endo)phenotypes across the TBI spectrum (10, 23). Even mild TBI can cause endothelial stress (24) and increase systemic levels of inflammatory molecules, including interleukin-6 (IL-6) (25). Many (if not most) moderate and all severe TBI patients suffer additional injuries to internal organs, muscles, and bones (i.e., polytrauma) (26). Polytrauma creates hugely complex systemic pathobiological responses (27) and damage to the cerebral vasculature that enables the cellular and humoral components of the inflammatory response to access the brain parenchyma (20, 28). The effect of such abnormal intracranial chemo- and cytokine milieu on the recovery process after TBI is not well understood. Importantly, this is a two-way street as illustrated by, for example, heterotopic ossification: the conversion of muscle tissue into bone which occurs in a subset of patients who also sustain TBI in addition to bone fractures (29). While the exact mechanism is unknown, soluble factors originating from the injured brain appear to alter the regenerative process and convert muscle cells into bone (30), illustrating the crosstalk between various organs affected by physical insults.

### Cofactor, Comorbidities

The importance of cofactors, age, biological sex and co-morbidities have been amply demonstrated in the outcomes of COVID-19 (31) and are also known in TBI (32–35). However, the overwhelming majority of experimental studies model TBI in young male rats ignoring the pediatric and elderly populations, the fastest growing segment, that together account for up to ~80% of all TBI cases (34). TBI causes very different biological responses in young versus old individuals who typically have comorbidities and related medications (36–38). History of COVID-19 can change the individual's biological background with major implications from diagnosis to treatment in TBI (11, 39). Biological sex greatly impacts outcome after TBI (32, 40) but females are currently hugely under-represented in experimental and clinical studies despite existing knowledge about the effects of hormones on TBI-induced pathobiological responses such as inflammation (41).

## Technical

### Big Data

From drug and vaccine design (42, 43) to vaccine distribution (44), Big Data (BD), Artificial Intelligence, and Machine Learning have been playing a critical role in the fight against the pandemic (reviewed by) (45). C3.ai (https://c3.ai/products/c3-ai-covid-19-data-lake/) created a COVID-19 specific “data lake,” a huge, analysis ready COVID-19 data repository (at no charge!), while Palantir (https://www.palantir.com/pt_media/palantir-is-providing-coronavirus-monitoring-to-the-cdc/) is providing COVID-19 monitoring, contact tracing, vaccine manufacturing and distribution. The ability to analyze huge amounts of unformatted and heterogeneous data would be especially critical for TBI (46–48). The TBI field has already made the critical first steps into BD by establishing the Federal Interagency Traumatic Brain Injury Research Informatics System (FITBIR) (https://fitbir.nih.gov; August 31,2020) using Common Data Elements (CDE) established for both preclinical and clinical TBI data (49, 50). Integrating FITBIR with large, biomedical data lakes that contain data from diseases other than TBI and all pathological conditions will enable better understanding of the myriad TBI-induced molecular pathologies. For instance, the cytokine IL-6 is the same molecule in COVID-19 and in TBI, with similar downstream signaling pathways and similar activators. But the importance lies is the context in which this cytokine is found. Numerous databases containing searchable data of various aspects of inflammation, anti-inflammatory molecules, molecular signaling, and disease phenotypes are available and can be queried for molecular networks using systems biology approaches (51, 52). BD approaches in TBI have been hampered by insufficient volumes of data and lack of data standards (46, 47, 53), but the benefits of using BD can be transformational in identifying pharmacological and other therapies for TBI-induced disorders.

### Drug Repurposing

One of the great uses of BD approaches to combat COVID-19 is in identifying drugs that target specific pathobiological processes triggered by SARS-CoV-2 infection (54). The Lancet lists ongoing clinical trials (55) and maps the evidence-based network of COVID-19 trials for the top 15 interventions. Candidate molecules include nucleotide analogs (Remdesivir) originally developed to treat Ebola (54), anti-inflammatory steroids (dexamethasone), monoclonal antibodies (mAb) (Camrelizumab and Tocilizumab, respectively) developed to treat Hodgkin's disease and rheumatoid arthritis (56, 57) as well as traditional Chinese medicines that activate the body's own repair and regenerative capacity (58). It has been recognized that drug repurposing is the fastest way to identify pharmacotherapies for TBI-induced pathobiologies (59). Many of the pathobiological processes induced by the injury have been identified (6) and much can be learned about the molecular targets using databases of other disorders. Many of those repurposed drugs have been successful in experimental studies but failed in clinical trials likely due to missing the therapeutic windows. Translating therapeutic windows from rodent to human is another challenge for BD (60).

### Novel Therapies

mRNA-based vaccines and mAb-based immunotherapies have been game changers in the fight against COVID-19. mAb therapies have also been used to treat CNS disorders including multiple sclerosis and Alzheimer's Disease with varying degrees of success (61, 62). Altered IL-6 signaling is an important component of the TBI-induced inflammatory response (63), and existing mAbs and/or antibody cocktails targeting IL-6 signaling can be repurposed to mitigate the TBI-induced inflammatory process (56). Furthermore, a new non-inflammatory mRNA vaccine has been successful in treating experimental autoimmune encephalomyelitis (64). Autoimmunity likely plays an important role in determining long-term outcome after TBI (65–67). The new mRNA-based therapy appears to control autoreactive T cells without inducing systemic immune suppression (64), which is currently a major hurdle in treating autoimmune disorders. While mAb- and mRNA-based treatments can be exciting and powerful therapies in treating some of the TBI-induced pathologies, such precision medicines require similarly precise knowledge about the identity of their molecular targets and critically, their therapeutic windows.

### Concluding Remarks

Experimental and clinical TBI research has generated vast amounts of data during the last several decades. Global clinical frameworks, TEAM TBI, CENTER-TBI, and now the China registry, provide the foundation for better understanding the biology of the disease, improving diagnostics, and developing evidence-based treatments through global collaborations. Learning from other diseases about their underlying molecular pathologies and pharmacological targets can greatly improve our understanding of TBI-induced pathobiological responses and identifying efficient treatment strategies. “Lessons learned” from COVID-19, including the importance of the temporal dimension of TBI-induced disease processes could accelerate integrating existing and novel data, guide drug repurposing and the development of novel, high-precision pharmacotherapies.

## Author Contributions

The author confirms being the sole contributor of this work and has approved it for publication.

## Conflict of Interest

The author declares that the research was conducted in the absence of any commercial or financial relationships that could be construed as a potential conflict of interest.

## Publisher's Note

All claims expressed in this article are solely those of the authors and do not necessarily represent those of their affiliated organizations, or those of the publisher, the editors and the reviewers. Any product that may be evaluated in this article, or claim that may be made by its manufacturer, is not guaranteed or endorsed by the publisher.
